# Mandatory Infant Vaccinations in France During the COVID-19 Pandemic in 2020

**DOI:** 10.3389/fped.2021.666848

**Published:** 2021-05-28

**Authors:** Marion Taine, Lucile Offredo, Jérôme Drouin, Julie Toubiana, Alain Weill, Mahmoud Zureik, Rosemary Dray-Spira

**Affiliations:** ^1^EPI-PHARE (French National Agency for Medicines and Health Products Safety, ANSM; and French National Health Insurance, CNAM), Saint-Denis, France; ^2^Department of General Paediatrics and Paediatric Infectious Diseases, Necker-Enfants Malades Hospital, AP-HP, Université de Paris, Paris, France

**Keywords:** COVID-19 pandemic, vaccination, infants (birth to 2 years), surveillance, national

## Abstract

**Objectives:** To describe changes in the dispensation of 11 mandatory vaccines to infants in France during the COVID-19 pandemic in 2020, considering the priming doses and boosters separately.

**Methods:** With data from the French national health database, all dispensations of priming doses and boosters of 11 mandatory vaccines [penta/hexavalent, measles mumps rubella (MMR), meningococcal conjugate type-C (Men-C-C), 13-valent pneumococcal conjugate (PCV13)] for infants ≤24 months old were aggregated by 4-week periods in 2020. Expected counts in 2020 were estimated according to counts in 2019 weighted by a ratio considering the level of vaccine dispensation before the pandemic onset in 2020. Relative differences (RDs) and their 95% confidence intervals (CIs) were computed to compare the observed and expected counts during the first and second lockdown and the period in between.

**Results:** During the first 4 weeks of the first lockdown, as compared with the expected numbers, the observed priming dose counts substantially decreased [RD: from −5.7% (95% CI −6.1; −5.2) for penta/hexavalent to −25.2% (95% CI −25.6; −24.8) for MMR], as did the booster counts [RD: from −15.3% (95% CI −15.9; −14.7) for penta/hexavalent to −20.7% (95% CI −21.3; −20.2) for Men-C-C]. Counts for priming doses and boosters remained slightly below the expected numbers after the lockdown. During 2020, MMR priming doses and the Men-C-C booster had the greatest shortfalls (*N* = 84,893 and 72,500, respectively).

**Conclusions:** This study provides evidence of a lack of vaccination catch-up after the first lockdown and a persistent shortfall in infant vaccination after the first 10 months of the COVID-19 pandemic in France, especially for the MMR priming doses and Men-C-C booster.

## Introduction

A mandatory immunization program is critical to protect infants against potentially life-threatening infections, and its schedule needs to be carefully respected (1), especially for the priming doses (2, 3). Experts consider that any delay in these priming doses is dangerous for the infant, whereas a delay of 1 or 2 months may be relativized for boosters (2, 4). The coronavirus disease (COVID-19) pandemic and its consequences have potentially disturbed this immunization program (5–8).

The COVID-19 pandemic was declared in France in February 2020 and has rapidly threatened the hospital system with saturation. France implemented its first national lockdown from March 17 to May 10, 2020, stopping non-essential economic and educational activities (9). This measure allowed for the attenuation of the first wave of the COVID-19 pandemic (10). With the acceleration of the second wave starting in August 2020, France implemented a curfew from October 17–30, 2020, then a second lockdown (October 30 to December 15), followed by a national curfew, which is ongoing. Although usual medical care access was maintained, parents may have been conflicted between the need to have their infant vaccinated and the fear that the infant might become infected by attending the practitioner's office (11).

Our aim was to describe the changes in the dispensation of 11 mandatory vaccines [measles, mumps, rubella (MMR), meningococcal conjugate type-C (Men-C-C), 13-valent pneumococcal conjugate (PCV13), and diphtheria, tetanus, pertussis, poliomyelitis, haemophilus influenzae type b conjugate with or without hepatitis B combined in the hexavalent or pentavalent vaccine] (12, 13) among infants during the COVID-19 pandemic in France in 2020, considering the priming doses and boosters separately.

## Methods

In France, the infant immunization program is administered in primary care at ages 2, 4, and 11 months for the penta/hexavalent and PCV13 vaccines, 5 and 11 months for Men-C-C, and 12 and 16 months for MMR (13). Priming doses are defined as the first dose for Men-C-C and one of the first two doses for the other vaccines. The second dose for Men-C-C and the third dose for penta/hexavalent and PCV13 vaccines are the boosters. The MMR vaccine does not require a booster during infancy.

Analyses were conducted with data from the French National Health Data System, which contains all healthcare claims reimbursement data for 98.8% of the French population (14). The counts of reimbursed dispensations of each vaccine among infants ≤24 months old in 2020 were aggregated by 4-week periods. For each 4-week period from February 17 onward, expected counts in 2020 were estimated by weighting the number of dispensations observed in 2019 by the ratio of observed counts over weeks two to seven in 2020 to the corresponding counts in 2019. This process allowed for the consideration of the increasing trend of vaccine coverage between 2019 and 2020 before the onset of the pandemic in France (15). Relative differences (RDs) and their 95% confidence intervals (CIs) were computed to compare the observed and expected counts during the following periods: before (February 17–March 15) and during the first lockdown (March 16–May 10), then between the two lockdowns (May 11–October 29), and during the second lockdown (October 30–December 20). Every user of French public health insurance is informed of their right to oppose the use of their data for research purposes.

## Results

### Evolution of Dispensed Priming Doses During the COVID-19 Pandemic

**Figure 1:**
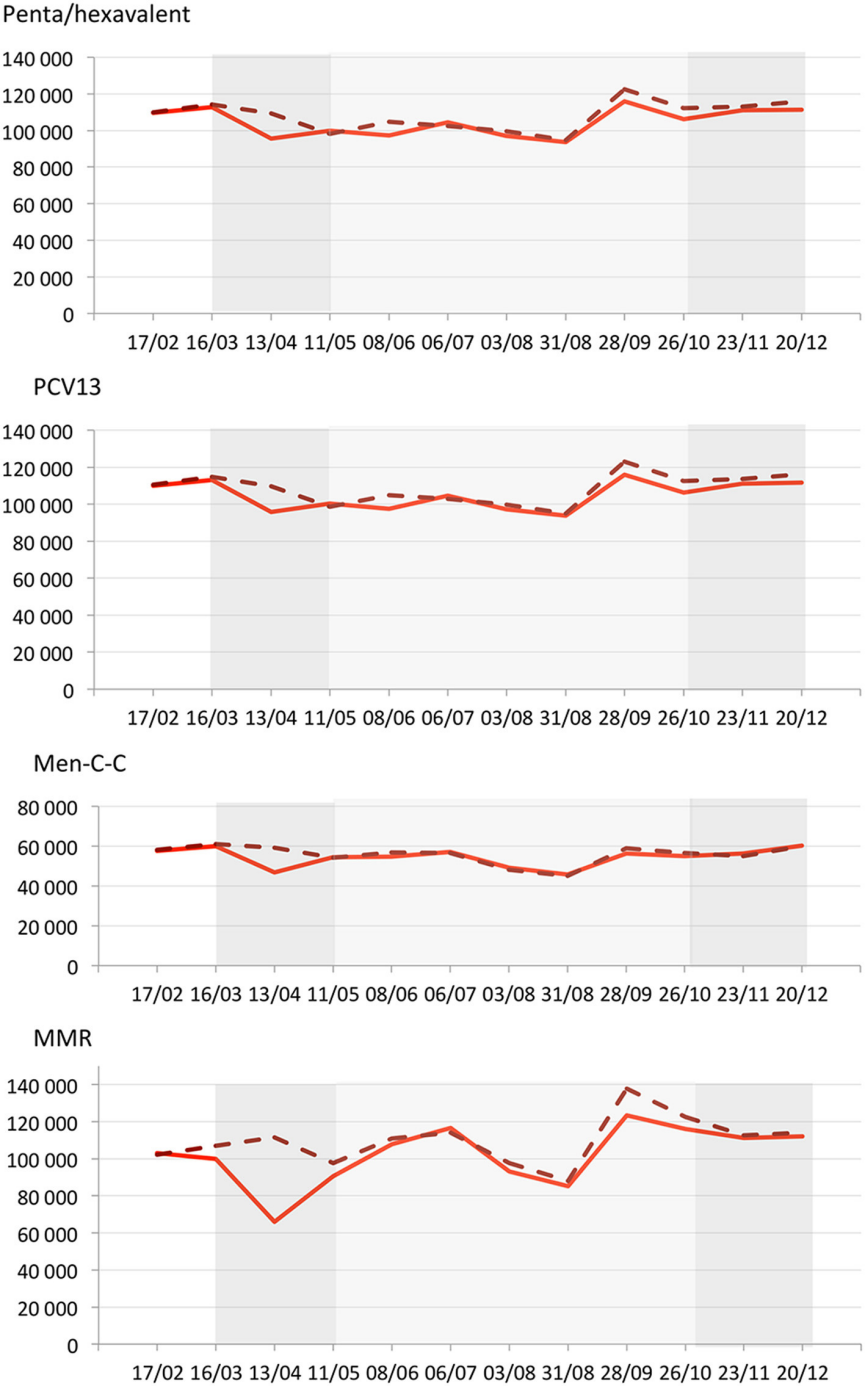
Observed (solid red line) and expected (dashed red line) weekly counts for priming dose dispensations of penta/hexavalent, 13-valent-pneumococcal conjugate (PCV13), meningococcal-conjugate type-C (Men-C-C) and measles mumps rubella (MMR) in France (from January to December 2020). Dark gray rectangles illustrate the first and the second lockdowns (from 16/03 to 10/05 and from 30/10 to 15/12, respectively). Light gray rectangle illustrates the inter-lockdown period (11/05 to 29/10). The X-axis is in 4-week periods since the beginning of 2020. The Y axis represents the number of dispensed priming doses over the 4 previous weeks at each time point.

During the 4 weeks before the first lockdown, the number of priming dose dispensations was slightly below what was expected [penta/hexavalent: RD −1.3% (95% CI −1.9; −0.7); PCV13: −1.5% (95% CI −2.1; −0.9); Men-C-C: −1.7% (95% CI −2.5; −0.9); MMR: −6.5% (95% CI −7.1; −5.9); Figure 1 and Table 1]. During the first 4 weeks of the lockdown, as compared with the expected numbers, the observed priming dose counts decreased by 12.4% (95% CI 11.9; 13.0) for penta/hexavalent, 12.6% (95% CI 12.0; 13.1) for PCV13, 21.0% (95% CI 20.3; 21.7) for Men-C-C, and 40.9% (95% CI 40.4; 41.3) for MMR. Then, the number of priming dose dispensations gradually increased during the second part of the lockdown, before slightly fluctuating below what was expected in the following 2 months. During the summer, counts were close to the expected numbers and slightly decreased again in September and October [penta/hexavalent: RD −5.4% (95% CI −5.8; −5.0); PCV13 −5.6% (95% CI −6.0; −5.2); Men-C-C: −3.7% (95% CI −4.2; −3.1); MMR −8.0% (95% CI −8.4; −7.6)]. Thus, during the 24 weeks after the first lockdown, the number of priming dose dispensations remained slightly lower than what was expected [penta/hexavalent: RD −3.4% (95% CI −3.6; −3.1); PCV13 −3.6% (95% CI −3.9; −3.4); Men-C-C: −1.3% (95% CI −1.7; −1.0); MMR −4.3% (95% CI −4.5; −4.1)].

**Table 1:** Relative differences between the observed and expected priming dose counts for penta/hexavalent, 13-valent pneumococcal conjugate (PCV13), meningococcal conjugate type C (Men-C-C), and measles mumps rubella (MMR) vaccines during the COVID-19 pandemic in France in 2020.

	**Penta/hexavalent vaccine**^**a**^			**PCV13 vaccine**			**Men-C-C vaccine**			**MMR vaccine**		
	**Observed**	**Expected**	**Relative difference**	**Observed**	**Expected**	**Relative difference**	**Observed**	**Expected**	**Relative difference**	**Observed**	**Expected**	**Relative difference**
	***n***	***n***	**% [95% CI]**	***n***	***n***	**% [95% CI]**	***n***	***n***	**% [95% CI]**	***n***	***n***	**% [95% CI]**
**Pre-lockdown** Feb 17-March 15	**112,716**	**114,213**	**−1.3 [−1.9;** **−0.7]**	**112,990**	**114,698**	**−1.5 [−2.1;** **−0.9]**	**60,113**	**61,138**	**−1.7 [−2.5;** **−0.9]**	**100,067**	**107,056**	**−6.5 [−7.1;** **−5.9]**
**First lockdown**	**195,636**	**207,387**	**−5.7 [−6.1;** **−5.2]**	**196,234**	**208,334**	**−5.8 [−6.2;** **−5.4]**	**101,234**	**113,622**	**−10.9 [−11.5;** **−10.4]**	**156,637**	**209,409**	**−25.2 [−25.6;** **−24.8]**
March 16-April 12	95,667	109,233	−12.4 [−13; −11,9]	95,904	109,706	−12.6 [−13.1; −12]	46,861	59,301	−21 [−21.7; −20.3]	65,969	111,613	−40.9 [−41.3; −40.4]
April 13-May 10	99,969	98,154	1.8 [1.2;2.5]	100,330	98,628	1.7 [1.1;2.4]	54,373	54,321	0.1 [−0.7;0.9]	90,668	97,796	−7.3 [−7.9; −6.7]
**Inter-lockdown**	**614,658**	**636,199**	**−3.4 [−3.6;** **−3.1]**	**615,340**	**638,407**	**−3.6 [−3.9;** **−3.4]**	**317,816**	**322,104**	**−1.3 [−1.7;** **−1]**	**642,213**	**671,009**	**−4.3 [−4.5;** **−4.1]**
May 11-June 7	97,379	104,694	−7 [−7.6; −6.4]	97,470	105,015	−7.2 [−7.8; −6.6]	54,696	56,787	−3.7 [−4.5; −2.9]	107,753	111,065	−3 [−3.6; −2.4]
June 8-July 5	104,431	102,558	1.8 [1.2;2.4]	104,495	102,984	1.5 [0.9;2.1]	57,081	56,690	0.7 [−0.1;1.5]	116,573	114,056	2.2 [1.6;2.8]
July 6-Aug 2	97,068	99,605	−2.5 [−3.2; −1.9]	97,131	99,798	−2.7 [−3.3; −2.1]	49,190	48,202	2.1 [1.2;3]	93.021	97,600	−4.7 [−5.3; −4.1]
Aug 3–30	93,697	94,623	−1 [−1.6; −0.3]	93,792	94,975	−1.2 [−1.9; −0.6]	45,707	45,059	1.4 [0.5;2.4]	85,327	87,904	−2.9 [−3.6; −2.3]
Aug 31-Sept 27	115,914	122,602	−5.5 [−6; −4.9]	116,063	122,996	−5.6 [−6.2; −5.1]	56,264	58,878	−4.4 [−5.2; −3.6]	123,470	137,823	−10.4 [−10.9; −9.9]
Sept 28-Oct 25	106,169	112,117	−5.3 [−5.9; −4.7]	106,389	112,639	−5.5 [−6.1; −5]	54,878	56,488	−2.8 [−3.7; −2]	116,069	122,561	−5.3 [−5.8; −4.8]
**Second lockdown**	**222,409**	**228,968**	**−2.9 [−3.3;** **−2.5]**	**222,745**	**230,088**	**−3.2 [−3.6;** **−2.8]**	**116,474**	**115,191**	**1.1 [0.5;1.7]**	**223,193**	**226,520**	**−1.5 [−1.9;** **−1.1]**
Oct 26-Nov 23	110,950	113,126	−1.9 [−2.5; −1.3]	110,996	113,754	−2.4 [−3; −1.8]	56,321	55,058	2.3 [1.5;3.1]	111,199	112,530	−1.2 [−1.8; −0.6]
Nov 24-Dec 20	111,459	115,842	−3.8 [−4.3; −3.2]	111,749	116,334	−3.9 [−4.5; −3.4]	60,153	60,133	0 [−0.8;0.8]	111,994	113,990	−1.8 [−2.3; −1.2]
**Since first lockdown**March16-Dec 20	**1,032,703**	**1,072,554**	**−3.7 [−3.9;** **−3.5]**	**1,034,319**	**1,076,829**	**−3.9 [−4.1;** **−3.8]**	**535,524**	**550,917**	**−2.8 [−3.1;** **−2.5]**	**1,022,043**	**1,106,936**	**−7.7 [−7.8;** **−7.5]**

a *Pentavalent and hexavalent vaccines combine vaccines for diphtheria, tetanus, pertussis, poliomyelitis, haemophilus influenzae type b conjugate without or with hepatitis B, respectively*.

*CI, confidence interval*.

During the 8 weeks of the second lockdown, the number of priming dose dispensations remained close to what was expected [penta/hexavalent: RD −2.9% (95% CI −3.3; −2.5); PCV13 −3.2% (95% CI −3.6; −2.8); Men-C-C: 1.1% (95% CI 0.5; 1.7); MMR: −1.5% (95% CI −1.9; −1.1)]. Overall, between March 16 and December 20, 2020, the number of priming dose dispensations was lower than what was expected for all vaccines, with shortfalls reaching 39,851 doses of penta/hexavalent [RD −3.7% (95% CI −3.9; −3.5)], 42,510 of PCV13 [−3.9% (95% CI −4.1; −3.8)], 15,393 of Men-C-C [−2.8% (95% CI −3.1; −2.5)], and 84,893 of MMR [−7.7% (95% CI −7.8; −7.5)]. Of note, ~70% of the shortfalls for penta/hexavalent and PCV13 priming doses occurred after the first lockdown.

### Evolution of Dispensed Boosters During the COVID-19 Pandemic

**Figure 2:**
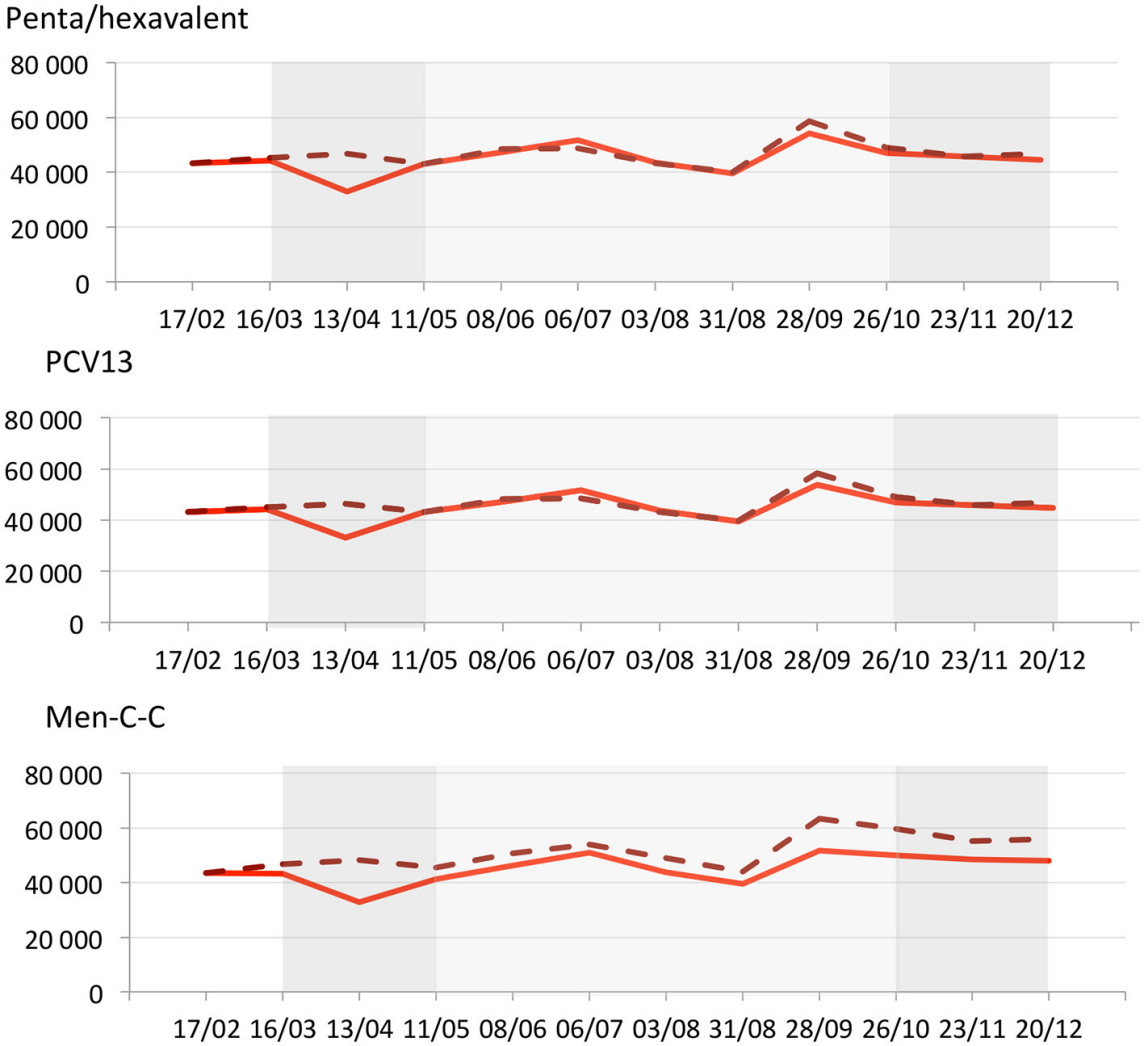
Observed (solid red line) and expected (dashed red line) weekly counts for booster dispensations of penta/hexavalent, 13-valent-pneumococcal conjugate (PCV13), meningococcal-conjugate type-C (Men-C-C) and measles mumps rubella (MMR} in France (from January 20 to December 20, 2020). Dark gray rectangles illustrate the first and the second lockdowns (from 16/03 to 10/05 and from 30/10 to 15/12, respectively). Light gray rectangle illustrates the inter-lockdown period (11/05 to 29/10). The X-axis is in 4-week periods since the beginning of 2020. The Y axis represents the number of dispensed boosters over the 4 previous weeks at each time point.

For boosters, in the 4 weeks before the first lockdown, the number of dispensations was slightly below what was expected [penta/hexavalent: RD −2.4% (95% CI −3.3; −1.5); PCV13: −1.7% (95% CI −2.6; −0.8); Men-C-C: −7.7% (95% CI −8.5; −6.8); Figure 2 and Table 2]. During the first 4 weeks of the lockdown, as compared with the expected numbers, observed booster counts decreased by 29.4% (95% CI 28.6; 30.2) for penta/hexavalent, 28.7% (95% CI 28.0; 29.5) for PCV13, and 31.9% (95% CI 31.2; 32.7) for Men-C-C. Then, the number of booster dispensations returned to levels close to those expected for all boosters. During the 24 weeks after the first lockdown, booster dispensations remained slightly below those expected for penta/hexavalent and PCV13 [RD −1.7% (95% CI −2.1; −1.4) and −1.5% (95% CI −1.9; −1.1), respectively] but substantially reduced for Men-C-C [RD −11.9% (95% CI −12.2; −11.6)]. During the 8 weeks of the second lockdown, the number of booster dispensations was similarly reduced [penta/hexavalent: RD −2.6% (95% CI −3.2; −1.9); PCV13 −2.3% (95% CI −3.0; −1.7); Men-C-C: −13.4% (95% CI −13.9; −12.9)]. Overall, the number of booster dispensations between March 16 and December 20, 2020 was lower than what was expected for all vaccines, with shortfalls reaching 21,140 doses of penta/hexavalent [RD −4.5% (95% CI −4.8; −4.2)], 19,700 of PCV13 [−4.2% (95% CI −4.5; −3.9)], and 72,500 of Men-C-C [−13.8% (95% CI −14.1; −13.5)].

**Table 2:** Relative differences between the observed and expected booster counts for penta/hexavalent, 13-valent pneumococcal conjugate (PCV13), and meningococcal conjugate type-C (Men-C-C) vaccines during the COVID-19 pandemic in France in 2020.

	**Penta/hexavalent vaccine**^**a**^			**PCV13 vaccine**			**Men-C-C vaccine**		
	**Observed**	**Expected**	**Relative difference**	**Observed**	**Expected**	**Relative difference**	**Observed**	**Expected**	**Relative difference**
**Pre-lockdown** Feb 17–March 15	**44,174**	**45,262**	**−2.4 [−3.3;** **−1.5]**	**44,218**	**44,996**	**−1.7 [−2.6;** **−0.8]**	**43,218**	**46,813**	**−7.7 [−8.5;** **−6.8]**
**First lockdown**	**76,037**	**89,782**	**−15.3 [−15.9;** **−14.7]**	**76,278**	**89,536**	**−14.8 [−15.4;** **−14.2]**	**74,164**	**93,552**	**−20.7 [−21.3;** **−20.2]**
March 16-April 12	32,945	46,666	−29.4 [−30.2; −28.6]	33,123	46,472	−28.7 [−29.5; −28]	32,776	48,152	−31.9 [−32.7; −31.2]
April 13-May 10	43,092	43,116	−0.1 [−1;0.9]	43,155	43,064	0.2 [−0.7;1.2]	41,388	45,400	−8.8 [−9.7; −8]
**Inter-lockdown**	**283,135**	**288,153**	**−1.7 [−2.1;** **−1.4]**	**282,693**	**286,971**	**−1.5 [−1.9;** **−1.1]**	**282,285**	**320,478**	**−11.9 [−12.2;** **−11.6]**
May 11-June 7	47,149	48,541	−2.9 [−3.7; −2]	47,164	48,295	−2.3 [−3.2; −1.5]	46,254	50,640	−8.7 [−9.5; −7.8]
June 8-July 5	51,618	48,661	6.1 [5.2;7]	51,630	48,463	6.5 [5.6;7.5]	50,885	53,871	−5.5 [−6.4; −4.7]
July 6-Aug 2	43,575	43,243	0.8 [−0.2;1.7]	43,618	43,104	1.2 [0.2;2.1]	43,872	48,945	−10.4 [−11.2; −9.5]
Aug 3–30	39,593	40,013	−1.1 [−2; −0.1]	39,499	39,744	−0.6 [−1.6;0.4]	39,598	44,047	−10.1 [−11; −9.2]
Aug 31-Sept 27	54,194	58,673	−7.6 [−8.4; −6.9]	53,874	58,382	−7.7 [−8.5; −6.9]	51,826	63,328	−18.2 [−18.9; −17.5]
Sept 28-Oct 25	47,006	49,022	−4.1 [−5; −3.2]	46,908	48,983	−4.2 [−5.1; −3.4]	49,850	59,647	−16.4 [−17.2; −15.7]
**Second lockdown**	**90,212**	**92,589**	**−2.6 [−3.2;** **−1.9]**	**90,453**	**92,617**	**−2.3 [−3;** **−1.7]**	**96,386**	**111,305**	**−13.4 [−13.9;** **−12.9]**
Oct 26-Nov 23	45,690	45,777	−0.2 [−1.1;0.7]	45,779	45,734	0.1 [−0.8;1]	48,358	55,255	−12.5 [−13.3; −11.7]
Nov 24-Dec 20	44,522	46,812	−4.9 [−5.8; −4]	44,674	46,883	−4.7 [−5.6; −3.8]	48,028	56,050	−14.3 [−15.1; −13.5]
**Since first lockdown**March 16-Dec 20	**449,384**	**470,524**	**−4.5 [**–**4.8;** –**4.2]**	**449,424**	**469,124**	**−4.2 [−4.5;** **−3.9]**	**452,835**	**525,335**	**−13.8 [−14.1;** **−13.5]**

a *Pentavalent and hexavalent vaccines combine vaccines for diphtheria, tetanus, pertussis, poliomyelitis, haemophilus influenzae type b conjugate without or with hepatitis B, respectively*.

## Discussion

During the first 10 months of the COVID-19 pandemic in 2020 in France, all mandatory priming dose and booster dispensations were reduced as compared with the expected estimates based on the previous year. The reduction was particularly striking during the first 4 weeks of the first lockdown, especially for the MMR priming doses and the Men-C-C booster. Since August 2020, the second wave of the pandemic was associated with a slight continual deficit in vaccine dispensations affecting the priming doses and boosters in similar proportions, and these trends were not substantially affected by the second lockdown.

Early decreases in infant vaccination uptake in France during the first lockdown appear to be less marked than in the United States (6–8) but higher than in the United Kingdom (5), where routine vaccinations are not mandatory. After the first month of the lockdown, the shortfall attenuated somewhat faster than in the United States (6–8). French authorities' messages promoting infant vaccination, which were widely disseminated from the third week of the lockdown, may have contributed to this (16–18). As in the United States, (6–8) our study in France reveals that during the first lockdown, the decrease was somewhat less prominent for the priming doses usually administered before 5 months of age (penta/hexavalent, PCV-13, Men-C-C priming doses) than for boosters and MMR, which are administered at an older age.

During the immediate post-lockdown period, the counts for all mandatory vaccine dispensations remained slightly below what was expected. In August 2020, no clear trend toward a catch-up had occurred. Since the onset of the second wave, vaccine dispensations have been declining again. This second deficit differed from the pattern during the first wave: priming doses and boosters were slightly and persistently affected in similar proportions (about 4%).

During the first 10 months of the COVID-19 pandemic, vaccines with suboptimal coverage in the previous years, namely the Men-C-C booster and MMR priming dose, were more affected than the others (15). For these two vaccines, the cumulative shortfalls reached more than 70,000 doses since the pandemic onset. Also, for the penta/hexavalent and PCV13 priming doses, 70% of the cumulative shortfalls occurred after the first lockdown. From a Delphi survey, experts considered that a delay >15 days for most of the priming doses and a delay >2 months for the MMR priming dose and boosters after the recommended age are potentially dangerous for infants (2).

The chronic vaccine deficit might also cause new outbreaks of vaccine-preventable diseases. Following the mandatory Men-C-C vaccination, the coverage of the Men-C-C first dose increased from 39% in 2017 to 76% in 2018 (15) and led to a decrease in invasive meningococcal C infections in 2019 (invasive meningococcal C infection incidence was 0.1/100,000 inhabitants in France in 2019) (19). However, the waning immunity due to the deficit of Men-C-C booster at 12 months of age may put infants who did not receive the booster at risk of invasive meningococcal C infection after 1 year of age (20). For the MMR vaccine, the first dose coverage at 12 months of age increased by only 3.3 points in France between 2016 and 2018 (from 74.3 to 77.7%) (15). Given the insufficient vaccine coverage of the overall population, measles outbreaks were observed in recent years in the United States and in several European countries including France (the measles incidence was 4/100,000 inhabitants in 2019) (21–23). Although barrier measures and the two lockdowns slowed the overall outbreaks of respiratory diseases, the MMR vaccine deficit observed in 2020 might favor new measles outbreaks after the COVID-19 pandemic. Recently, resurgences of pertussis were noted among the youngest children in the United States and several European countries despite a high vaccine coverage (24). Several hypotheses are under consideration to explain these resurgences, such as an earlier waning of protective immunity of the pertussis acellular vaccine and the circulation of *Bordetella* pertussis variants with depletion of vaccine-included antigens (24). In this context, the prolonged vaccination deficit, albeit slight, might promote new pertussis outbreaks.

The vaccine shortfall may be explained by parental concerns about potentially exposing their children to COVID-19 during the immunization visit (5, 6). Parental knowledge about the benefits of the first vaccinations for their infants may have mitigated their fears and limited the decrease in delivery of these priming doses during the first month of the pandemic. Beyond the fear of COVID-19, vaccination delay or refusal may be related to vaccine hesitancy (25), especially in France, with the strongest negative sentiment related to vaccine safety in the world (26). A systematic review showed that barriers to vaccination access were persistent among children from families of low socioeconomic status (27). Risk factors for vaccination shortfalls, especially socio-economic and geographic factors, need to be explored to identify infants at risk of vaccine delay.

This study has substantial strengths. It is based on a comprehensive national database from a country with ~1,400,000 infants born per year (28). To better assess the potential influence of the pandemic and its consequences on vaccine dispensations, the expected counts were calculated considering the increasing trend of vaccine coverage (15) in early 2020 as compared with 2019. We separately investigated the priming dose and boosters because a delay in the priming doses is more dangerous than for the boosters.

The main limitation of this study is that the evolution of dispensed vaccinations does not precisely provide information on the status of vaccine coverage in France. These dispensation data may have underestimated the deficit of actually injected vaccines during the pandemic because parents may have obtained the vaccine at the pharmacy but not visited the practitioner for injection for fear of exposing their child to COVID-19 (11). Finally, the administrative nature of the data does not provide information on the causes of the lack of vaccine catch-up and the chronic lack of vaccine dispensations.

## Conclusion

This study provides evidence of a persistent shortfall in infant vaccination during the first 10 months of the COVID-19 pandemic in France, especially for the MMR priming doses and Men-C-C booster. The increasing trend of vaccine coverage observed since 2018 (15) has been weakened as a consequence of the pandemic in France. Vaccination should be closely monitored and strongly encouraged using all available media to safeguard children against future outbreaks.

## Data Availability Statement

The datasets presented in this article are not readily available because the procedures carried out with the French data privacy authority (CNIL, Commission nationale de l'informatique et des libertés) do not provide for the transmission of the database. All requests for access must be submitted to the Health data hub. Requests to access the datasets should be directed to these websites: https://www.snds.gouv.fr/SNDS/Processus-d-acces-aux-donnees—https://documentation-snds.health-data-hub.fr/introduction/03-acces-snds.html#les-acces-sur-projet.

## Ethics Statement

Ethical review and approval was not required for the study on human participants in accordance with the local legislation and institutional requirements. Written informed consent from the participants' legal guardian/next of kin was not required to participate in this study in accordance with the national legislation and the institutional requirements.

## Author Contributions

AW, RD-S, MZ, and MT conceptualized and designed the study. JD and LO designed the data collection instruments, collected data, and carried out the initial analyses. MT supervised data collection and drafted the initial manuscript. AW, RD-S, MZ, JD, LO, and JT critically reviewed the manuscript for important intellectual content. All authors approved the final manuscript as submitted and agreed to be accountable for all aspects of the work.

## Conflict of Interest

The authors declare that the research was conducted in the absence of any commercial or financial relationships that could be construed as a potential conflict of interest.
